# Graph Optimization Model Fusing BLE Ranging with Wi-Fi Fingerprint for Indoor Positioning

**DOI:** 10.3390/s22114045

**Published:** 2022-05-26

**Authors:** Rong Zhou, Puchun Chen, Jing Teng, Fengying Meng

**Affiliations:** School of Control and Computer Engineering, North China Electric Power University, Beijing 102206, China; zhourong@ncepu.edu.cn (R.Z.); 120202227078@ncepu.edu.cn (P.C.); 120202227098@ncepu.edu.cn (F.M.)

**Keywords:** indoor positioning, Wi-Fi fingerprint, BLE ranging, graph optimization

## Abstract

To improve the user’s positioning accuracy of a Wi-Fi fingerprint-based positioning algorithm, this study proposes a graph optimization model based on the framework of $g^{2}$o that fuses a Wi-Fi fingerprint and Bluetooth Low Energy (BLE) ranging technologies. In our model, the improvement in positioning can be formulated as a nonlinear least-squares optimization problem that a graph can represent. The graph regards users as nodes and our self-designed error functions between users as edges. In the graph, the nodes obtain the initial coordinates through Wi-Fi fingerprint positioning, and all error functions aggregate to a total error function to be solved. To improve the solution effect of the total error function and weaken the influence of measurement error, an information matrix, an edge selection principle, and a Huber kernel function are introduced. The Levenberg–Marquardt (LM) algorithm is used to solve the total error function and the affine transformation estimation is used for the drifting solution. Through experiments, the influence of the threshold in the Huber kernel function is explored, the relationship between the number of nodes in the graph and the optimization effect is analyzed, and the impact of the distribution of nodes is researched. The experimental results show improvements in the positioning accuracy of four common Wi-Fi fingerprint-matching algorithms: KNN, WKNN, GK, and Stg.

## 1. Introduction

With increasing demand for location-based services (LBSs), indoor positioning has become a research topic of much interest [1]. In recent years, some RF-based methods for precise indoor positioning have emerged, mainly in two categories: trilateration [2] and fingerprint [3]. The trilateration method requires the physical distance between the target point and at least three anchor points. The measurements used to calculate physical distance in trilateration include received signal strength indication (RSSI) [4], time difference of arrival (TDOA) [5], and time of arrival (TOA) [6]. Compared to TDOA and TOA, RSSI-based ranging avoids the need for expensive clock synchronization systems and additional hardware, and has the advantages of low cost, easy deployment, and simple calculation. The fingerprint method is regarded as superior to trilateration in more complex indoor environments, where the interference, reflection, and refraction of signal forces are superimposed onto the specific points [7].

Wi-Fi technology is widely used in indoor target intrusion detection and localization [8,9]. The Wi-Fi wireless access points (APs) have been widely deployed in indoor environments because of the improvement of wireless sensor technology and the growing demand for network communication, and they provide a physical device foundation for the application of Wi-Fi fingerprint positioning. The Wi-Fi fingerprint method is regarded as low-cost and simple because it requires no additional hardware and performs satisfactorily in complex indoor environments [10]. However, room exists for improvement in its positioning accuracy in complex indoor environments such as underground parking lots.

Multi-sensor fusion positioning is a common method to improve positioning accuracy. Fusing BLE and Wi-Fi for positioning inherits their advantages in low costs and the widespread deployment of basic equipment. Bluetooth Low Energy (BLE) products can run for months on a battery with low-power communication. Its transmission distance of up to 50–100 m meets the requirements of indoor positioning for wireless signals [11,12]. BLE technology has been embedded in billions of products, such as mobile phones, earphones, computers, and cars, providing the basis for its application in indoor positioning. Subsequent studies using BLE beacons for indoor positioning [13] provide a theoretical basis for our work.

Multiple users can use smart devices to obtain initial positioning coordinates by the Wi-Fi fingerprint method. Because BLE signals from other devices are available everywhere, the physical distance between users can be measured by BLE ranging technology and can act as additional constraints to adjust user coordinates.

We propose a graph optimization model combining a Wi-Fi fingerprint and BLE ranging technology to improve indoor positioning accuracy. The general process is as follows. The problem can be considered a nonlinear least-squares optimization problem that a graph can represent, where users are regarded as nodes, and our designed error functions between users are edges. We obtain the initial coordinates of nodes based on the Wi-Fi fingerprint method and aggregate all edges to a total error function. The information matrix, edge selection principle, and Huber kernel function are used to control the measurement error of wireless sensors. The Levenberg–Marquardt (LM) algorithm can solve the total error function to obtain the optimized coordinates of the nodes, and the affine transformation estimation can solve the drifting problem of optimized nodes.

The remainder of this paper is organized as follows. Section 2 introduces the related theories of the Wi-Fi fingerprint method, signal propagation of BLE, and graph optimization based on $g^{2}o$. Section 3 provides an overview of the proposed model, the error function and total error function, the drifting solution, BLE signal processing, and the BLE ranging model. Section 4 describes our experimental process and results from multiple perspectives and verifies the feasibility and generalization of the proposed graph optimization model. Section 5 summarizes our study, draws conclusions, and suggests further work.

## 2. Related Work

### 2.1. Wi-Fi Fingerprint Positioning

Many well-known indoor positioning systems have been developed based on the Wi-Fi fingerprint method, such as RADAR [14], Nibble [15], and Horus [16]. Proposed fingerprint-matching algorithms include the classic k-nearest neighbors (KNN) [17] and WKNN [18], which assigns weights to each reference point based on KNN. The other two matching algorithms (Stg and GK) used in the experimental part of this paper are introduced as follows.

The Stg algorithm [19] selects the strongest AP data in the initial sample filtering, which can not only reduce the size of the fingerprint database, but can also reduce the calculation speed of the fingerprint-matching process. The fingerprint-matching process of Stg algorithm is based on the KNN algorithm. The GK algorithm [20] is based on parametric modeling of a logarithmic RSS stochastic process, in which each RSS is considered to be the mean, and the standard deviation is set as a constant for all observations. The probability value of the test fingerprint at each RP is calculated by the Gaussian kernel density estimator, and the position of the test point is estimated according to the average value of the coordinates of several RPs with the highest probability value.

**Figure 1:**
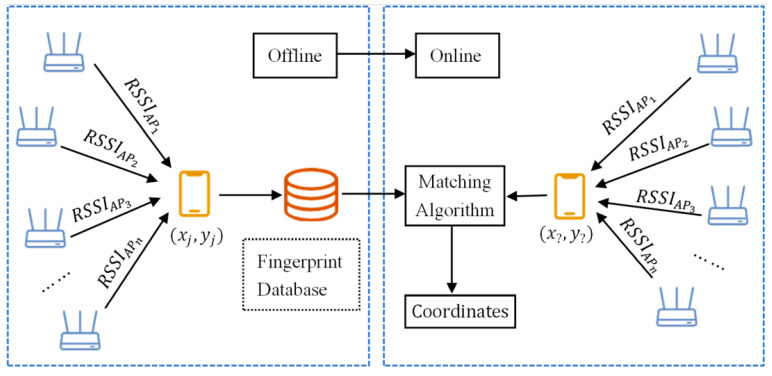
General process of Wi-Fi fingerprint positioning.

Neural network and deep-learning technologies are also applied in Wi-Fi fingerprint positioning [21,22], but regardless of the system or algorithm, the Wi-Fi fingerprint method always follows a general process consisting of offline and online phases, as shown in Figure 1.

In the offline phase, a certain number of APs are deployed, several reference points (RPs) are set up evenly in the indoor environment, and the RSSIs from APs at the RPs are collected to establish a fingerprint database. A fingerprint consists of the physical coordinates and Wi-Fi signal fingerprints of the RPs. Suppose the number of APs involved in positioning is n, the number of RPs is m, and $RSSI_{\left(i,j\right)}$ represents the RSSI from $AP_{i}$ received at $RP_{j}$. Since the RSSI is easily affected by environmental changes, multiple RSSI collections are carried out at each RP to build a fingerprint database; so, the *k*th fingerprint at $RP_{j}$ can be expressed as

(1) $$FP_{j}^{\left(k\right)}=\left[RSSI_{\left(1,j\right)}^{\left(k\right)},RSSI_{\left(2,j\right)}^{\left(k\right)},\ldots ,RSSI_{\left(n,j\right)}^{\left(k\right)},x_{j},y_{j}\right]$$

If we collect fingerprints k times at $RP_{j}$, the Wi-Fi fingerprint database (WFPDB) can be saved as a matrix:

(2) $$\mathrm{WFPDB}=\left(\begin{array}{c} FP_{1}^{\left(1\right)},FP_{1}^{\left(2\right)},\ldots ,FP_{1}^{\left(k\right)}\\ FP_{2}^{\left(1\right)},FP_{2}^{\left(2\right)},\ldots ,FP_{2}^{\left(k\right)}\\ \ldots \\ FP_{m}^{\left(1\right)},FP_{m}^{\left(2\right)},\ldots ,FP_{m}^{\left(k\right)} \end{array}\right)$$

In the online phase, we estimate the positions of users by collecting the RSSIs of different APs in real-time and applying the fingerprint-matching algorithm.

### 2.2. Signal Propagation of BLE

Common propagation path-loss models include the free space propagation and logarithmic distance path-loss models [23]. The latter is generally used in BLE ranging technology [24,25] and is expressed as

(3) $$P_{t}\left(d\right)=P_{l}\left(d_{0}\right)-10\lambda \mathrm{lg}\left(\frac{d}{d_{0}}\right)+\varepsilon ,$$

where $P_{t}\left(d\right)$ is the RSSI when the distance between the Bluetooth transmitting point and receiving point is d, $d_{0}$ is the reference distance, $P_{l}\left(d_{0}\right)$ is the RSSI when the distance between the transmitting and receiving point is $d_{0}$, ε is Gaussian random noise, and λ is the path loss index. For convenience of calculation, usually, $d_{0}$ = 1 m. To fit the signal propagation model based on the collected RSSI, the simplified model can be expressed as

(4) RSSI=A−10λlg(d),

where $\mathrm{RSSI}=P_{t}\left(d\right)$ and $A=P_{l}\left(1\right)$. Both A and λ are empirical values that are related to the indoor environment. By collecting the RSSI of BLE beacons at different distances and fitting the parameters by least-squares, Equation (4) can be used for BLE ranging.

### 2.3. Graph Optimization Based on $g^{2}$o

Many robotics and computer vision studies involve the minimization of nonlinear error functions, a typical example being simultaneous localization and mapping (SLAM) [26,27,28]. These studies aim to find parameters or state variables that best explain a set of measured values affected by Gaussian noise, where the LM algorithm is used to find the optimal solution [29].

**Figure 2:**
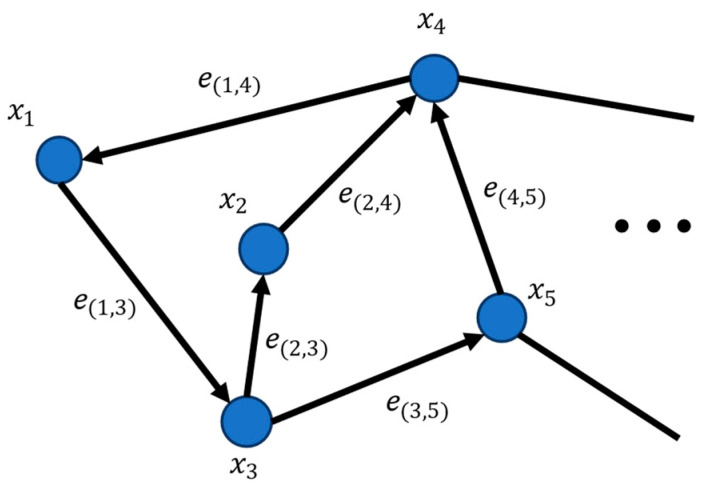
Graphical representation of nonlinear least-squares problems.

$g^{2}$o is a general back-end optimization framework suitable for nonlinear least-squares problems [30], which can be represented graphically in the framework of $g^{2}$o, as shown in Figure 2, where each node represents a state variable to be optimized, and an edge between nodes represents a paired observation of the two nodes.

Graph optimization based on $g^{2}$o represents a nonlinear least-squares problem as a graph and visually displays the structural relationship between the variables. The graph can be directed or undirected, and the corresponding optimization problem can be described as

(5) $$F\left(x\right)=e_{\left(1,3\right)}^{T}\Omega_{\left(1,3\right)}e_{\left(1,3\right)}+e_{\left(2,3\right)}^{T}\Omega_{\left(2,3\right)}e_{\left(2,3\right)}+e_{\left(2,4\right)}^{T}\Omega_{\left(2,4\right)}e_{\left(2,4\right)}+e_{\left(3,5\right)}^{T}\Omega_{\left(3,5\right)}e_{\left(3,5\right)}+e_{\left(4,5\right)}^{T}\Omega_{\left(4,5\right)}e_{\left(4,5\right)}+\ldots ,$$

where F(x) is the final total error function to be solved, $e_{\left(i,j\right)}$ is the error function between nodes $x_{i}$ and $x_{j}$, and $\Omega_{\left(i,j\right)}$ is the information matrix of edge $e_{\left(i,j\right)}$. Using BLE ranging data to optimize Wi-Fi fingerprint location results can be abstracted as a graph optimization problem. Users can be defined as nodes in the graph, and the definition of edges between nodes is introduced in Section 3.2.

## 3. Proposed Graph Optimization Model

### 3.1. Model Overview

**Figure 3:**
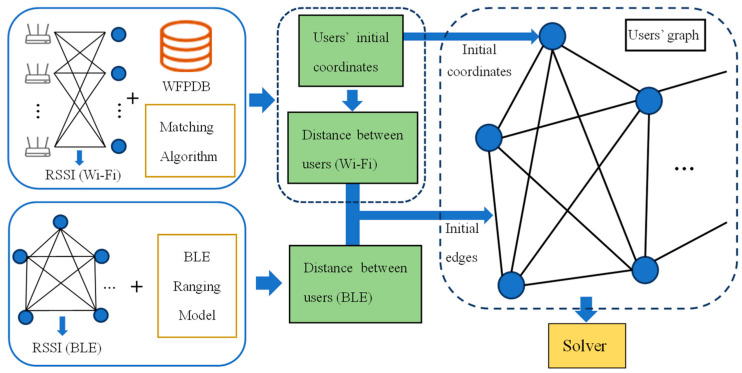
Construction of the proposed model.

Wi-Fi fingerprint positioning can provide position results at the current moment that are not dependent on the previous one, so there is no cumulative error as the user moves. However, coordinates obtained in this way are not accurate enough due to noise. To optimize the positioning results of a Wi-Fi fingerprint, physical distances between users measured by BLE ranging technology are added to establish redundant constraints. Then, graph optimization based on $g^{2}$o can be adopted to adjust the coordinates of users in the graph and improve positioning accuracy. The construction of the model is shown in Figure 3.

We define the users as nodes of the graph and obtain their initial positioning coordinates by the construction of an offline Wi-Fi fingerprint database and online fingerprint matching. Users receive BLE RSSIs from other users’ devices and input them to the BLE ranging model to obtain physical distances to other users. We define the error function and regard it as the edges between pairs of nodes. The total error function is constructed by all edges and grows in size with the number of users. The optimal solution of the total error function is obtained by the solver, i.e., the LM algorithm and the drifting solution introduced in Section 3.3.

There will inevitably be several edges with large relative errors in the graph that affect optimization. The robust kernel function, the method of reconstructing the information matrix introduced in Section 3.2, and the filtering conditions of the edges determined by the experiments in Section 4.2 are introduced to weaken the effects of these edges. 

When we experimented with the proposed model, we used a laptop (Lenovo, Beijing, China) running Windows 10 as the central server for user positioning calculation. The users need to provide the RSSIs received from APs to the central server, and the initial coordinates of the users can be obtained based on the Wi-Fi fingerprint positioning. The RSSIs received from other users’ devices with BLE function also need to be input into server to run the graph optimization model, and users can obtain the optimized position from the server.

### 3.2. Construction and Solution of Total Error Function

Constructing a reasonable total error function is the key to solving the graph optimization problem. As the number of user nodes in the graph increases, the number of variables $\left(x_{i}\;\mathrm{and}\;y_{i}\right)$ in the total error function expand twice as fast as nodes. We find that the number of nodes grows linearly, while the number of edges grows exponentially in the graph as the number of users joining the positioning increases. Because the total error function can easily fall into a locally optimal solution during iteration, assigning each node a good initial value can effectively solve the above problems, that is, obtaining the user’s initial coordinates through the Wi-Fi fingerprint positioning. The graph, nodes, and edges are defined as follows.

Suppose there are n user nodes in the graph, user node i is defined as $p_{i}=\left(x_{i},y_{i}\right),\left(1\leq i\leq n\right)$, and its initial value as obtained by Wi-Fi fingerprint positioning is denoted as $p_{i}^{w}=\left(x_{n}^{w},y_{n}^{w}\right),\left(1\leq i\leq n\right)$. The distance obtained by the BLE ranging model between nodes i and j is defined as $d_{\left(i,j\right)}^{b},\left(1\leq i,j\leq n,\;i\neq j\right)$, and the distance between nodes i and j in the graph is defined as

(6) $$d_{\left(i,j\right)}=\sqrt{{\left(x_{i}-x_{j}\right)}^{2}+{\left(y_{i}-y_{j}\right)}^{2}},\left(i\neq j\right).$$

Since the distance between users measured by the BLE ranging model adds constraints on nodes, in our proposed model, $d_{\left(i,j\right)}$ will be close to $d_{\left(i,j\right)}^{b}$ by adjusting the locations of nodes. So, the edge between $p_{i}$ and $p_{j}$ is defined as

(7) $$e\left(p_{i},p_{j}\right)=d_{\left(i,j\right)}^{b}-d_{\left(i,j\right)},\left(1\leq i,j\leq n\right),\left(i\neq j\right).$$

The total error function of the entire graph is composed of all of the error functions (called edges) between pairs of nodes. It can be defined as

(8) $$F\left(p\right)=\sum_{\langle p_{i},p_{j}\rangle \in p}e^{T}\left(p_{i},p_{j}\right)\Omega_{\left(i,j\right)}e\left(p_{i},p_{j}\right),$$

(9) $$p^{*}=\arg\min F\left(p\right),$$

where $\Omega_{\left(i,j\right)}$ is the information matrix of $e\left(p_{i},p_{j}\right)$, and $p=\left\{p_{1},p_{2},\ldots ,p_{n}\right\}$ and $p^{*}=\left\{p_{1}^{*},p_{2}^{*},\ldots ,p_{n}^{*}\right\}$ are respectively a set of user nodes before and after optimization. $p_{i}^{*}=\left(x_{i}^{*},y_{i}^{*}\right),\left(1\leq i\leq n\right)$ is the final position of $p_{i}$ after optimization, because $p_{i}$ keeps changing during the iterative solution of the total error function.

After defining the nodes and edges, we can define the size of the information matrix as 1×1, which can be regarded as a weighting coefficient. The distance error between nodes based on the Wi-Fi fingerprint positioning cannot be predicted accurately; so, we do not consider adding the statistical indicators of Wi-Fi fingerprint positioning to calculate the information matrix.

Previous experiments with the BLE ranging model have shown that the RSSI of BLE can be approximated to a Gaussian distribution for simplicity [31]. As the distance increases, the BLE RSSI fluctuation increases, which directly leads to an increase in the fluctuation of the ranging error. The relationship between true distance and BLE ranging distance can be defined as

(10) $$d_{\left(i,j\right)}^{b}=d_{\left(i,j\right)}^{r}+l_{\left(i,j\right)},$$

where $d_{\left(i,j\right)}^{r}$ is the real distance between $p_{i}$ and $p_{j}$, and $l_{\left(i,j\right)}$ is the error between $d_{\left(i,j\right)}^{b}$ and $d_{\left(i,j\right)}^{r}$. We use $d_{\left(i,j\right)}^{b}$ as a substitute for $d_{\left(i,j\right)}^{r}$ because $d_{\left(i,j\right)}^{r}$ cannot be predicted.

The experiments of Section 4.2 show that the distance error of BLE ranging is not only related to the physical distance, but is also closely related to the standard deviation (SD) of the ranging results. Thus, the SD of BLE ranging is added to calculate the information matrix:

(11) $$\Omega_{\left(i,j\right)}=\frac{1}{\sigma_{\left(i,j\right)}^{2}+1}.$$

For simplicity of notation, in the rest of this paper, we will express the error function in a new form:

(12) $$e\left(p_{i},p_{j}\right)\overset{def}{\Rightarrow }e_{\left(i,j\right)}\left(p\right).$$

The error function can be approximated by its first-order Taylor expansion around the current initial guess $\hat{p}$:

(13) $$\begin{array}{c} e\left({\hat{p}}_{i}+\Delta p_{i},{\hat{p}}_{j}+\Delta p_{j}\right)=e_{\left(i,j\right)}\left(\hat{p}+\Delta p\right)\\ \;\;\;\;\;\;\;\;\;\;\;\;\;\;\;\;\;\;\;\;\;\;\;\;\;\;\;\;\;\;\;\;\;\;\;\;\;\;\;\;≅e_{\left(i,j\right)}\left(\hat{p}\right)+J_{\left(i,j\right)}\Delta p,\; \end{array}$$

where $J_{\left(i,j\right)}$ is the Jacobian of $e_{\left(i,j\right)}\left(p\right)$, computed from the current estimate $\hat{p}$. Since there are errors in both BLE ranging and Wi-Fi fingerprint positioning, the graph will contain wrong edges in many cases. Nonlinear least-squares optimization is greatly affected by errors; thus, it is difficult to guarantee the convergence and performance of the total error function in the iterative process, even if the information matrix has been introduced to weaken the effect of errors. To reduce the occurrence of the above situation, the Huber kernel function [32] is introduced to adjust the edges.

When the error is small, the error function is approximated as a quadratic function with faster growth. When the error is large, it is approximated as a linear function with slower growth, which still ensures that the function is continuous and differentiable. The Huber kernel function is

(14) $$\rho_{H}\left(x\right)=\{\begin{array}{c} \;\;\;\;\frac{1}{2}x^{2},\;\;\left|x\right|<\delta \\ \delta \left|x\right|-\frac{1}{2}\delta^{2},\;\;\left|x\right|\geq \delta \end{array}\;,$$

and the total error function with the Huber kernel function can be rewritten as

(15) $$F\left(p\right)=\sum_{1\leq i,j\leq n}\rho_{H}\left(e_{\left(i,j\right)}\left(p\right)\right)\Omega_{\left(i,j\right)}.$$

To preserve the characteristics of the original total error function and the calculated parameters, we can simplify the processing of the Huber kernel function by adjusting the information matrix,

(16) $$\rho_{H}\left(e_{\left(i,j\right)}\left(p\right)\right)\Omega_{\left(i,j\right)}=e_{\left(i,j\right)}^{T}\left(p\right)\Omega_{\left(i,j\right)}^{U}e_{\left(i,j\right)}\left(p\right),$$

where $\Omega_{\left(i,j\right)}^{U}$ is the updated information matrix. The dimension of $e_{\left(i,j\right)}\left(p\right)$ is 1×1; thus,

(17) $$\rho_{H}\left(e_{\left(i,j\right)}\left(p\right)\right)\Omega_{\left(i,j\right)}={\left(e_{\left(i,j\right)}\left(p\right)\right)}^{2}\Omega_{\left(i,j\right)}^{U},$$

(18) $$\Omega_{\left(i,j\right)}^{U}=\frac{\rho_{H}\left(e_{\left(i,j\right)}\left(p\right)\right)\Omega_{\left(i,j\right)}}{{\left(e_{\left(i,j\right)}\left(p\right)\right)}^{2}}.$$

Substituting the updated information matrix in the original total error function, we obtain a new total error function with the original form:

(19) $$F_{H}\left(p\right)=\sum_{1\leq i,j\leq n}e_{\left(i,j\right)}^{T}\left(p\right)\Omega_{\left(i,j\right)}^{U}e_{\left(i,j\right)}\left(p\right).$$

Substituting Equation (13) in Equation (19), $F_{H}\left(\hat{p}+\Delta p\right)$ can be approximated as

(20) $$\begin{array}{c} \;\;\;F_{H}\left(\hat{p}+\Delta p\right)≅\underset{1\leq i,j\leq n}{\sum }{\left(e_{\left(i,j\right)}\left(\hat{p}\right)+J_{\left(i,j\right)}\Delta p\right)}^{T}\Omega_{\left(i,j\right)}^{U}\left(e_{\left(i,j\right)}\left(\hat{p}\right)+J_{\left(i,j\right)}\Delta p\right)\\ \;\;\;\;\;\;\;\;\;\;\;\;\;\;\;\;\;\;\;\;\;\;\;\;\;=\underset{1\leq i,j\leq n}{\sum }e_{\left(i,j\right)}^{T}\left(\hat{p}\right)\Omega_{\left(i,j\right)}^{U}e_{\left(i,j\right)}\left(\hat{p}\right)+2e_{\left(i,j\right)}^{T}\left(\hat{p}\right)\Omega_{\left(i,j\right)}^{U}J_{\left(i,j\right)}\Delta p\\ \;\;\;\;\;\;\;\;\;\;\;\;\;\;\;\;\;\;\;\;\;\;\;\;\;\;\;\;\;\;+\Delta p^{T}J_{\left(i,j\right)}^{T}\Omega_{\left(i,j\right)}^{U}J_{\left(i,j\right)}\Delta \;\;\;\;\\ \;\;\;\;\;\;\;\;\;\;\;\;\;\;\;\;\;\;\;\;\;\;\;\;\;=\underset{1\leq i,j\leq n}{\sum }c_{\left(i,j\right)}+2b_{\left(i,j\right)}\Delta p+\Delta p^{T}H_{\left(i,j\right)}\Delta p\\ \;\;\;\;\;\;\;\;\;\;\;\;\;\;\;\;\;\;\;\;\;\;\;\;\;=c+2b\Delta p+\Delta p^{T}H\Delta p.\;\;\;\;\;\;\;\;\;\;\;\;\;\;\;\;\;\;\;\;\;\;\;\;\;\;\;\; \end{array}$$

For simplicity of notation, we set $c_{\left(i,j\right)}=e_{\left(i,j\right)}^{T}\left(\hat{p}\right)\Omega_{\left(i,j\right)}^{U}e_{\left(i,j\right)}\left(\hat{p}\right)$, $b_{\left(i,j\right)}=e_{\left(i,j\right)}^{T}\left(\hat{p}\right)\Omega_{\left(i,j\right)}^{U}J_{\left(i,j\right)}$, $H_{\left(i,j\right)}=J_{\left(i,j\right)}^{T}\Omega_{\left(i,j\right)}^{U}J_{\left(i,j\right)}$, $c=\sum^{\;}c_{\left(i,j\right)}$, $b=\sum^{\;}b_{\left(i,j\right)}$, and $H=\sum^{\;}H_{\left(i,j\right)}$. $F_{H}\left(\hat{p}+\Delta p\right)$ can be minimized in Δp by solving the linear system based on the LM algorithm,

(21) $$\left(H+\alpha I\right)\Delta p^{*}=-b,$$

where H is the information matrix of this system, I is an identity matrix, and α is a damping factor. This is useful to control the step size in case of nonlinear surfaces and to control the convergence.

The solution is obtained by adding the increments $\Delta p^{*}$ to the initial guess:

(22) $$p^{*}=\hat{p}+\Delta p^{*}.$$

The popular LM algorithm iterates the linearization in Equation (20), the solution in Equation (21), and the update step in Equation (22). In every iteration, the previous solution is used as the linearization point and the initial guess until a given termination criterion is met. The derivation of the LM algorithm is not the work of this paper and is not repeated here. The implementation process of the LM algorithm is shown in Algorithm 1.
**Algorithm 1:** Pseudo code of LM algorithm   **Input:** A total error function *F_H_*(*p*) and an initial vector *p^w^*   **Output:** A vector p* minimizing *F_H_*(*p*) 

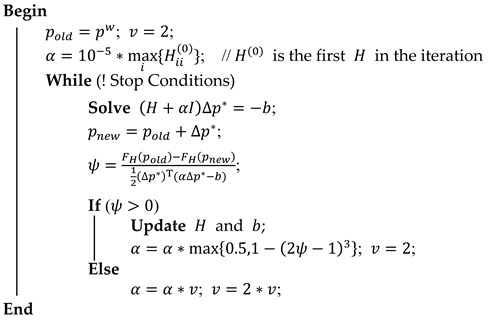



### 3.3. Drifting Solution Based on Affine Transformation Estimation

The optimized graph has a better structure, but the nodes may drift together because of the missing absolute coordinate constraints. Since the affine transformation estimation can relatively better preserve the optimized structure, we use it to solve the drifting problem. We define the nodes calculated by the LM algorithm as $p^{l}=\left(p_{1}^{l},p_{2}^{l},\ldots p_{n}^{l}\right)$ and the nodes estimated by affine transformation as $p^{t}=\left(p_{1}^{t},p_{2}^{t},\ldots p_{n}^{t}\right)$. We redefine the coordinates of each node as $p_{i}^{l}=\left(x_{i}^{l},y_{i}^{l},1\right),\;\left(1\leq i\leq n\right)$, $p_{i}^{t}=\left(x_{i}^{t},y_{i}^{t},1\right),\;\left(1\leq i\leq n\right)$ and $p_{i}^{w}=\left(x_{i}^{w},y_{i}^{w},1\right),\;\left(1\leq i\leq n\right)$. The transformation matrix is calculated by

(23) $$\{\begin{array}{c} p^{l}M=p^{w}\\ M=\left(\begin{array}{c} a,\;d,\;0\\ b,e,\;0\\ c,f,1 \end{array}\right) \end{array},$$

where M represents the transformation matrix. The optimal estimates of parameters a, b, c, d, e, and f in M are calculated by the least-squares method. Our final optimization results of the nodes are

(24) $$p^{t}=p^{l}M.$$

### 3.4. BLE Signal Filter and Ranging Model

**Figure 4:**
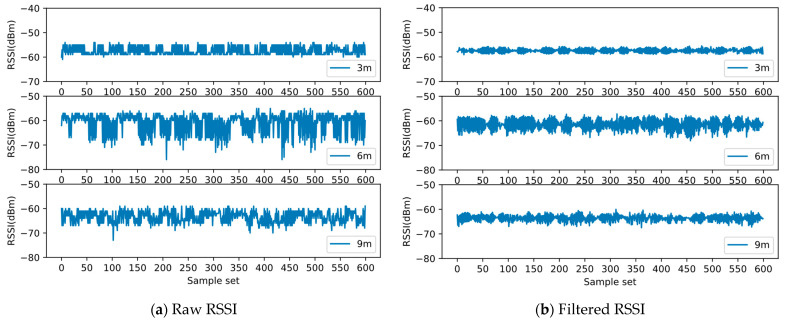
Raw and filtered received signal strength indication (RSSI) of Bluetooth Low Energy (BLE) at 3, 6, and 9 m.

During actual signal collection, the received RSSI from the BLE beacon fluctuates in the indoor environment. BLE ranging can affect the accuracy of the edges in the graph; thus, BLE ranging errors should be controlled within a certain range to build the network structure of nodes in the graph. In our experiments, the transmit power of the BLE beacon was +4 dBm, and its broadcast period was set to 80 ms. Figure 4a shows the RSSI data collected at 3, 6, and 9 m between the BLE beacon and the signal receiving point, and it can be observed that the original RSSI fluctuates greatly.

Since the broadcast period of the BLE beacon was set to 80 ms, the average number of RSSIs that could be collected every second was 12.5. To balance the positioning time and a reasonable filter window size, we adopted mean filtering and set the filter window to 10, that is, 10 raw BLE RSSIs were processed each time before input to the BLE ranging model. Obviously, this strategy can effectively reduce the signal fluctuation, as shown in Figure 4b.

Table 1 presents the comparison of the SD of RSSIs collected at 3, 6, and 9 m before and after filtering. The SD can be reduced by nearly 50% after filtering.

**Figure 5:**
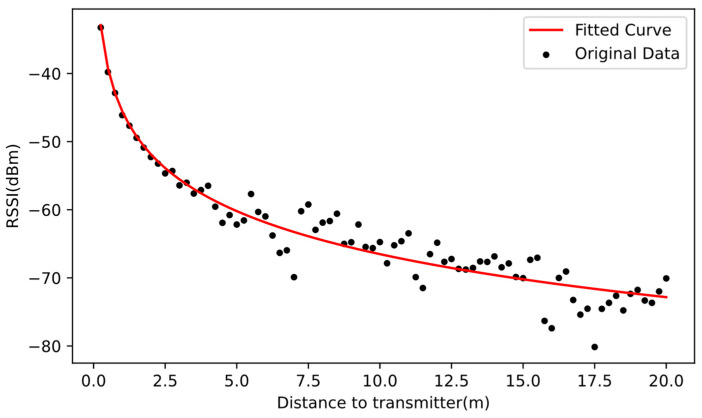
Fitted curve of BLE ranging model.

This paper uses the simplified BLE ranging model (Equation (4)) and estimates the values of A and n by the filtered RSSI at different distances (0.25 m interval) and least-square curve fitting, as shown in Figure 5.

Then, we can calculate the distance between user nodes based on this mode,

(25) RSSI=−45.688−20.835lg(d).

Through the linear fitting to the SD of the RSSI at different distances, we can obtain the signal propagation model applicable to our experiments,

(26) $$\{\begin{array}{c} RSSI=-45.688-20.835\mathrm{lg}\left(d\right)+\eta_{\left(i,j\right)}\\ \eta_{\left(i,j\right)}\sim N\left(0.24d_{\left(i,j\right)}^{r}+1.51\right) \end{array}.$$

## 4. Experiments

### 4.1. Experimental Environment

**Figure 6:**
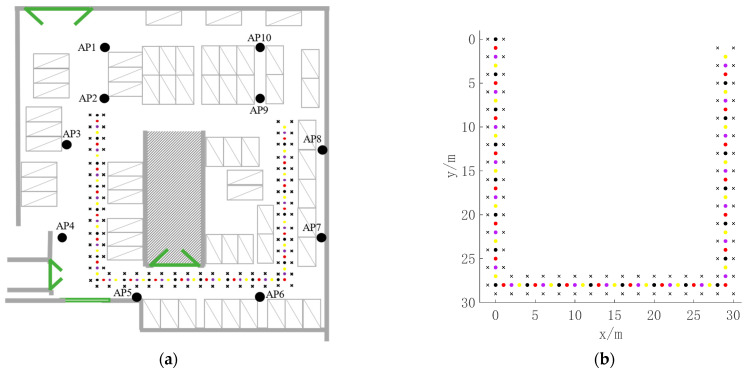
(**a**) Testbed plan; (**b**) coordinates of TPs and RPs.

Our experiments used the underground parking lot of North China Electric Power University as a testbed, where the Wi-Fi fingerprint database was constructed. The testbed was 58 m long and 42 m wide, and Wi-Fi APs were installed on the wall 2 m high relative to the parking lot surface. Figure 6a is a plan view of the testbed, where black dots mark the 10 AP locations. Test points (TPs) represent users, and RPs and TPs were set in the middle of the passage. The distance between two adjacent RPs was 2 m, and adjacent TPs were 1 m apart. We deployed 86 RPs and 84 TPs, where an “x” represents an RP and a four-color “o” represents a TP. Figure 6b shows the coordinates of RPs and TPs.

Our device was set 1 m high relative to the parking lot surface, collecting Wi-Fi data at each RP. We collected 10 sets of Wi-Fi data of 10 APs continuously at each RP to construct the Wi-Fi fingerprint database, and we used the Wi-Fi fingerprint-matching algorithm to obtain the initial location of each TP. To verify the validity of the proposed model and control the cost, we conducted experiments with simulated BLE ranging data and randomly selected points from 84 TPs to perform the graph optimization.

### 4.2. Edge Selection

**Figure 7:**
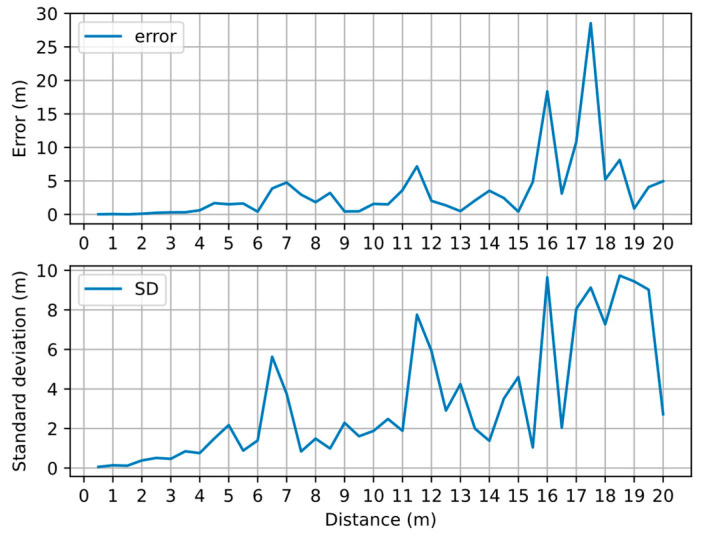
Error and standard deviation (SD) of BLE ranging at different distances.

Edges with larger errors in the graph are often formed by two user nodes that are far apart. Giving these edges a reasonable information matrix can weaken the influence of errors. However, when we calculate the information matrix $\Omega_{\left(i,j\right)}$ (Equation (11)), we need to determine the value of $\sigma_{\left(i,j\right)}$. We collected the BLE RSSIs every 0.5 m within a range of 20 m from the BLE beacon and input them to the BLE ranging model (Equation (25)) to calculate the error and SD of ranging in each collection point, as shown in Figure 7.

As the distance increases, the error and SD of ranging generally show a linear upward trend. The greater the distance, the more frequent the fluctuations of the error and SD. Experiments show that most BLE ranging errors within 15 m can be stably concentrated within 5 m, and more are concentrated within 2 m. Table 2 shows the ranging error in different distances within 15 m at 1 m intervals. In the experiment, we collected RSSIs at different distances and calculated the physical distance between the transmitting point and the receiving point, but the results show the discontinuity of the error. This is mainly due to the fact that the influence of the multipath effect on the ranging error is more significant than that of signal attenuation in the range of 20 m. Considering the relationship between ranging error and distance, we decided to only add edges within 15 m to the graph.

### 4.3. Influence of Threshold δ in Huber Kernel Function

The Huber kernel function can reduce the effect of large error edges in the graph. If the value of δ is set too small, the weight of most edges will be reduced, which will weaken the role of precise edges. If it is set too large, the weight of large error edges will not be reduced, which will cause the Huber kernel function to lose its effect. So, the δ value should be chosen appropriately.

**Figure 8:**
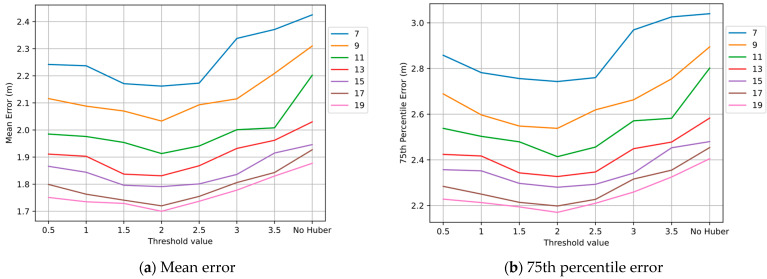
Positioning errors under different thresholds δ.

The experimental design of this part was as follows. We obtained the initial coordinates of 84 TPs by KNN matching algorithm and set the Huber kernel function threshold δ from 0.5 to 3.5. Seven control groups were set up with 7, 9, 11, 13, 15, 17, and 19 nodes per group. Each group randomly selected a corresponding number of points from 84 TPs for optimization and repeated the experiment 1000 times to obtain sufficient data. We also added a comparison experiment without the Huber kernel function in each group. The results in Figure 8 show that the mean positioning error and 75th percentile error were reduced by adjusting the value of threshold δ from 0.5 to 2. Adjusting the value of threshold δ from 2 to 3.5, the mean positioning error and 75th percentile error increased. Obviously, setting δ to 2 is reasonable.

### 4.4. Impact of Number of Nodes in Graph

**Figure 9:**
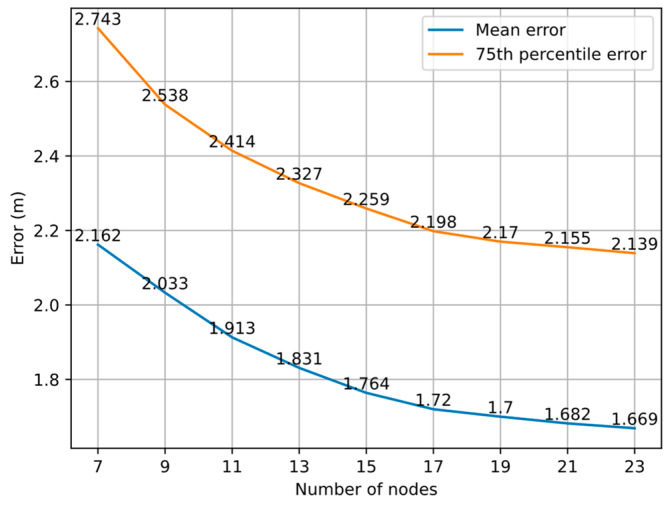
Positioning errors under different numbers of nodes.

In the experiment, we obtained the initial coordinates of 84 TPs based on the KNN algorithm and set the Huber kernel function threshold δ to 2. Nine control groups were set up with 7, 9, 11, 13, 15, 17, 19, 21, and 23 nodes per group. Each group randomly selected a corresponding number of points from 84 TPs for optimization and repeated the experiment 1000 times to obtain sufficient positioning coordinates. Figure 9 shows that the optimization effect improves as the number of nodes added to the graph for optimization increases from 7 to 23, and the magnitude of the improvement gradually decreases and the error curve tends to be steady.

**Figure 10:**
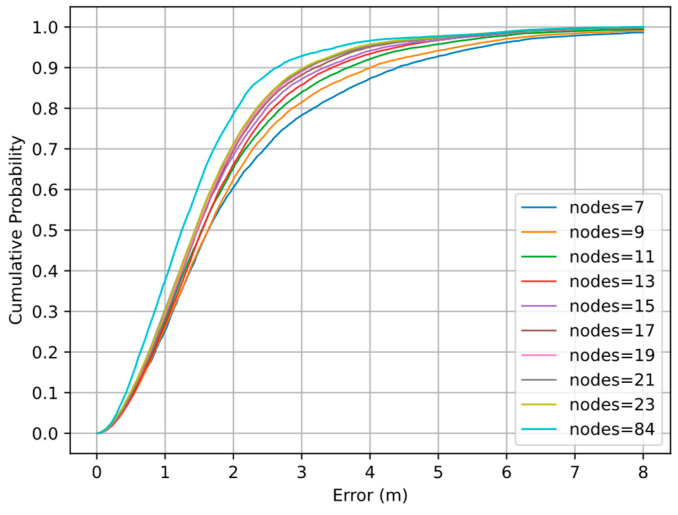
Positioning error cumulative distribution function (CDF) curves of different numbers of nodes.

When the number of nodes reaches 19, the reduction of the mean error and 75th percentile error is less than 2 cm for every two additional nodes. When the number of nodes is 7, the average distance between nodes is often greater than the threshold of 15 m, and too few edges can be added to the graph. Therefore, insufficient constraints and large errors will cause the failure of the total error function to converge and an improvement of positioning accuracy that is not obvious. When the number of nodes reaches 9, the total error function can converge in most cases, further improving the positioning accuracy. The experimental results show that under the KNN algorithm, we can obtain relatively stable positioning results when the number of nodes reaches 19 and δ is set to 2. Figure 10 shows the error cumulative distribution function (CDF) curves of different numbers of nodes. 

It can be observed that the spacing between the curves decreases as the number of nodes increases, and the curves with 19, 21, and 23 nodes are tightly packed together. The gap between the curves is especially obvious at 2–5 m, indicating that our graph optimization model can significantly improve the positioning accuracy at 2–5 m as the number of nodes increases.

### 4.5. Impact of Distribution of Nodes in Graph

The 84 TPs were labeled in U-shaped order with markers ranging from 1 to 84, which were sequentially divided into three groups. Each group contained 28 TPs, representing one of the three passages in the experimental area. We defined a good distribution as a random selection of nodes from only one of the three groups for graph optimization, and a bad distribution as a random and even selection of nodes from each of the three groups for graph optimization. In the experiment, we set the Huber kernel function threshold δ to 2 and separately conducted group experiments for two distributions. The number of nodes in each group was different and the experiment was repeated 1000 times per group.

**Figure 11:**
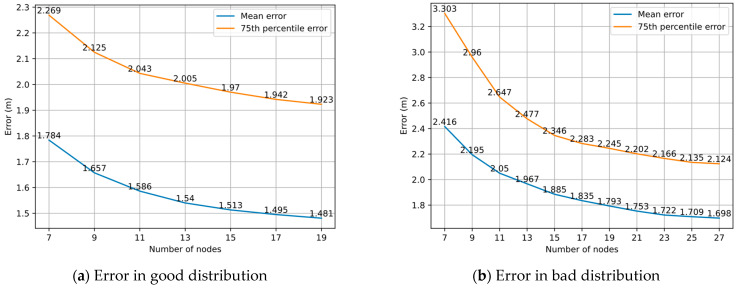
Positioning errors under different numbers of nodes in two types of distribution.

Figure 11a shows the positioning errors under different numbers of nodes in good distribution. It can be found that the mean errors and 75th percentile errors of the good distribution are lower than the random distribution (as shown in Figure 9) under the same number of nodes. The reduction of the mean error is less than 2 cm for every two additional nodes in 15 nodes, and the reduction of 75th percentile error is less than 2 cm for every two additional nodes in 17 nodes. Figure 11b shows the positioning errors under different numbers of nodes in bad distribution. In addition to the fact that the errors are higher than the random distribution, the reduction of the mean error and 75th percentile error is less than 2 cm for every two additional nodes in 23 nodes. In the bad distribution, the positioning accuracy decreases compared with the KNN algorithm when the number of nodes is 7, and the positioning error is only slightly lower than the KNN algorithm when the number of nodes reaches 9, which indicates that the number of nodes in our proposed model needs to be greater than or equal to 9 for better work in our experimental area.

The experimental results show that the error tends to level off with the node increase under both good and bad distributions, and the errors of a good distribution tend to level off faster than those of a bad distribution. This shows that our graph optimization model is influenced by the node distribution. We find that the better the node distribution, the higher the positioning accuracy for the same number of nodes. The positioning accuracy of a good distribution is more likely to achieve relatively stable optimization results.

### 4.6. Comparison of Wi-Fi Fingerprint-Matching Algorithms after Optimization

**Figure 12:**
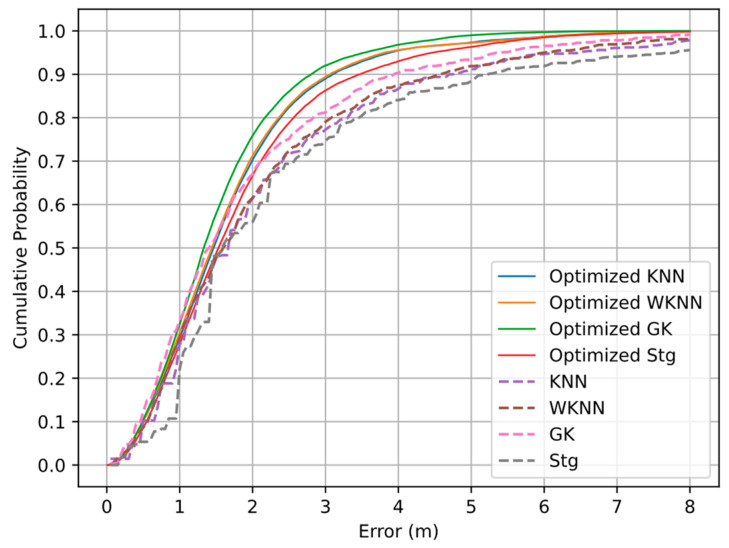
Positioning error CDF curves of Wi-Fi fingerprint-matching algorithms before and after optimization.

The proposed graph optimization model can be used to optimize Wi-Fi fingerprint-matching algorithms other than the KNN algorithm. In the experiments, we constructed the user graph with 19 random nodes and set the Huber kernel function threshold δ to 2 to optimize four Wi-Fi fingerprint-matching algorithms. The error CDF curves of the KNN, W-KNN, GK, and Stg algorithms before and after optimization are shown in Figure 12. Obviously, the proposed graph optimization model can improve the positioning accuracy. We find that the GK algorithm has the highest positioning accuracy, and the ranking of positioning effects before and after optimization has not changed.

The cumulative error probability of different algorithms under a fixed accuracy limit is shown in Table 3. It can be seen that the optimized algorithm (KNN, WKNN, and GK) does not dominate within 1 m, but the positioning effect is gradually improved from 1 m to 3 m, being most obvious at 3 m. This shows that our proposed algorithm can effectively correct node coordinates with large errors. The optimized Stg algorithm is slightly different at 1 m and 1.5 m, which is caused by the uneven error distribution of the Stg algorithm in the range of 0–2 m, as shown in Figure 12. The obvious optimized results at 2 m, 2.5 m and 3 m of the Stg algorithm again validated our conclusion.

Table 4 shows the optimization results of four Wi-Fi fingerprint-matching algorithms. In terms of the mean positioning error, the KNN algorithm improves the positioning accuracy by 23.1%, WKNN by 21.5%, GK by 19.4%, and Stg by 27.2%. The results show that the 75th percentile error and SD are also reduced after optimization, which shows the effectiveness of mixed BLE ranging and Wi-Fi fingerprint positioning based on the graph optimization model at improving the positioning accuracy. We find that the lower the initial localization accuracy, the more obvious the improvement of localization accuracy, which indicates that the proposed model works better for Wi-Fi matching algorithms with larger errors.

From the experimental results, it can be seen that the more accurate the initial positioning results output by Wi-Fi positioning, the more accurate the optimized positioning results, which indicates that the positioning results of our graph optimization model are sensitive to the initial positioning.

## 5. Conclusions and Further Work

We integrated Wi-Fi fingerprint positioning and BLE ranging technology to locate users in indoor environments based on a graph optimization model. After deriving the relationship between the physical distance and the RSSI of BLE, a BLE ranging simulation was carried out based on the actual RSSI standard deviation. We obtained initial positioning results through Wi-Fi fingerprint positioning, built a graph of users, added edges between user nodes based on BLE ranging data, and summarized them in a large-scale total error function, which we solved to obtain a better structure of nodes. A drifting solution based on the affine transformation was applied to obtain the final optimized position of the users. The experiments showed that the proposed graph optimization model has a relatively good positioning result when the kernel function threshold δ was set to 2. The reduction of the average positioning error and 75th percentile error was less than 2 cm for every two additional nodes when the number of nodes in the graph reached 19. Our graph optimization model can be influenced by the node distribution. The positioning accuracy of a good distribution is more likely to achieve relatively stable and accurate optimization results. The application of the proposed model to KNN, WKNN, GK, and Stg resulted in improvements, which shows that the proposed model is versatile. The positioning results of the four optimized Wi-Fi fingerprint-matching algorithms showed that the initial positioning results can affect the optimized positioning results.

Although the proposed model can improve the positioning accuracy of traditional Wi-Fi fingerprint positioning, there is room for improvement. The experiments showed that the positioning accuracy rankings of other Wi-Fi algorithms before and after optimization are basically the same, and they verified the effect of the initial positioning results on the optimization results. In addition, we hypothesize that the accuracy of ranging between the user nodes in the graph can affect the final positioning results. Therefore, our next step will be to go beyond the use of BLE beacons for ranging. For example, wireless sensors with higher accuracy can be used to construct more reliable edges of the user graph.

## Figures and Tables

**Table 1:** Standard deviation (SD) of received signal strength indication (RSSI) at 3, 6, and 9 m before and after filtering.

Distance (m)	SD (Original RSSI) (dBm)	SD (Filtered RSSI) (dBm)
3	1.802	0.935
6	4.553	2.594
9	2.542	1.498

**Table 2:** Ranging error within 15 m at 1 m intervals.

Distance (m)	Error (m)	Distance (m)	Error (m)
1	0.058	9	0.448
2	0.104	10	1.573
3	0.308	11	3.623
4	0.615	12	2.02
5	1.518	13	0.487
6	0.413	14	3.538
7	4.761	15	0.419
8	1.829		

**Table 3:** Cumulative error probability of different algorithms under fixed accuracy limit.

Algorithm	1 m	1.5 m	2 m	2.5 m	3 m
Optimized KNN	29.34%	51.73%	70.24%	82.35%	89.02%
Optimized WKNN	30.47%	52.72%	71.27%	82.79%	89.44%
Optimized GK	33.41%	57.88%	75.9%	86.04%	92.1%
Optimized Stg	27.93%	48.86%	66.59%	78.79%	86.26%
KNN	28.1%	47.86%	61.43%	71.55%	77.26%
WKNN	29.29%	47.74%	61.55%	72.38%	79.05%
GK	33.33%	52.62%	67.02%	75.12%	81.19%
Stg	22.14%	48.21%	55.95%	69.4%	74.64%

**Table 4:** Error of optimized Wi-Fi fingerprint-matching algorithms.

	Algorithm	Optimized KNN	Optimized WKNN	Optimized GK	Optimized Stg	KNN	WKNN	GK	Stg
Indicator									
Mean Error (m)		1.7	1.68	1.54	1.82	2.21	2.14	1.91	2.5
75th Percentile Error (m)		2.17	2.14	1.97	2.32	2.75	2.69	2.50	3.01
Error Std (m)		1.19	1.18	1.01	1.3	1.92	1.85	1.67	2.33

## Data Availability

The experiment uses an internal data set. The data presented in this study are available on request from the corresponding author.
